# Role of Phosphatidylinositol-3-Kinase Pathway in Head and Neck Squamous Cell Carcinoma

**DOI:** 10.1155/2012/450179

**Published:** 2012-05-16

**Authors:** Li Du, Jingping Shen, Andrew Weems, Shi-Long Lu

**Affiliations:** ^1^Department of Otolaryngology, University of Colorado, Anschutz Medical Campus, 12700 E 19th Avenue, Aurora, CO 80045, USA; ^2^Department of Otolaryngology, Shengjing Hospital of China Medical University, Shenyang 110004, China; ^3^Graduate Program in Cell Biology, Stem Cells and Development, University of Colorado, Anschutz Medical Campus, 12700 E 19th Avenue, Aurora, CO 80045, USA; ^4^Department of Dermatology, University of Colorado, Anschutz Medical Campus, 12700 E 19th Avenue, Aurora, CO 80045, USA; ^5^Department of Pathology, University of Colorado, Anschutz Medical Campus, 12700 E 19th Avenue, Aurora, CO 80045, USA

## Abstract

Activation of the phosphatidylinositol-3-kinase (PI3K) pathway is one of the most frequently observed molecular alterations in many human malignancies, including head and neck squamous cell carcinoma (HNSCC). A growing body of evidence demonstrates the prime importance of the PI3K pathway at each stage of tumorigenesis, that is, tumor initiation, progression, recurrence, and metastasis. Expectedly, targeting the PI3K pathway yields some promising results in both preclinical studies and clinical trials for certain cancer patients. However, there are still many questions that need to be answered, given the complexity of this pathway and the existence of its multiple feedback loops and interactions with other signaling pathways. In this paper, we will summarize recent advances in the understanding of the PI3K pathway role in human malignancies, with an emphasis on HNSCC, and discuss the clinical applications and future direction of this field.

## 1. Introduction

The phosphatidylinositol-3-kinase (PI3K) signaling pathway is one of the pathways most commonly activated in human cancers [1]. It is a major downstream signaling component of receptor tyrosine kinases (RTKs) and is critical for the regulation of cell proliferation, growth, differentiation, migration, and survival [2]. Thus, it represents one of the most promising targets for cancer prevention and therapy [3]. 

Mounting reports of original studies and reviews have been published, highlighting the paramount importance of this pathway in human cancers. To avoid redundancy with previous publications, in this paper we will focus on summarizing recent progress in PI3K pathway research in head and neck squamous cell carcinoma (HNSCC). Specifically, this will include considerations of the molecular alterations seen in some components of the PI3K pathway, as well as functional studies of the role of the PI3K pathway in HNSCC initiation, invasion, and metastasis studied both in vitro and in vivo, with a particular focus on the use of genetically engineered mouse models (GEMMs). Finally, we will explore the potential of the PI3K pathway as a target for chemoprevention and cancer therapy. 

## 2. Common Molecular Alterations of HNSCCs

HNSCC refers to squamous cell carcinomas (SCCs) arising from the oral cavity, tongue, pharyngeal, and laryngeal regions. As the 6th most common human cancer worldwide, they generate about 600,000 new cases and 350,000 cancer deaths each year [4, 5]. HNSCCs usually occur at a relatively late age and at a higher frequency in males possessing the well-known etiological factors of tobacco and/or alcohol usage [4, 5]. Recently, however, the incidence of HNSCC is increasing in women of a relatively young age, correlating with human papilloma virus (HPV) infection [4, 5]. 

Historically, the best known molecular alterations in HNSCC were the inactivation of tumor suppressors, such as p16 and p53, and activation of oncogenes, such as EGFR and Stat3 [5, 6]. We have studied the role of transforming growth factor beta (TGF*β*) pathway in HNSCC using both human HNSCC samples and GEMM approaches [7–10]. Our studies indicate that inactivation of the type II receptor of TGF*β* (TGF*β*RII) and the downstream signal mediator of TGF*β*, Smad4, plays a crucial role in HNSCC development and progression [8, 10]. Perhaps the most comprehensive studies on molecular alterations of HNSCC came from two recent publications describing whole exome sequencing on human HNSCC samples [11, 12]. Two major results were generated from these papers: (1) the discovery of novel molecular alterations of the Notch signaling pathway in human HNSCC samples and (2) the validation of the PI3K pathway as one of the major targets for molecular alterations in human HNSCC samples, including alterations of the oncogene PIK3CA and the tumor suppressor gene PTEN. These two papers, together with many previous reports, clearly demonstrate the importance of the PI3K pathway in HNSCC. Furthermore, they suggest its possible involvement in every aspect of HNSCC development and progression, including tumor initiation, invasion, recurrence, resistance to therapeutics, and metastasis.

## 3. PI3K Signaling Pathway

**Figure 1:**
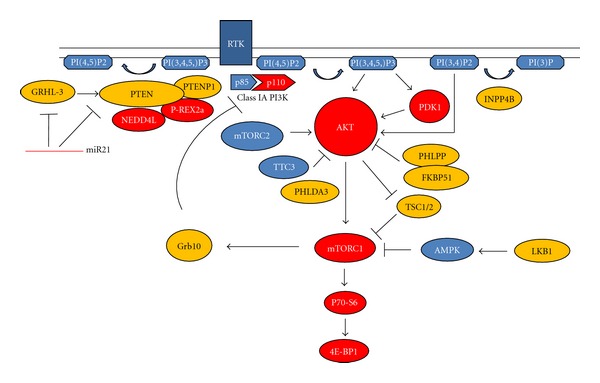
Schematic of the PI3K/AKT/mTOR pathway and its interacting molecules. Red: molecules have oncogenic property. Yellow: molecules have tumor suppression property.

PI3Ks are a family of enzymes that phosphorylate the 3-OH group on phosphatidylinositols. There are three classes of PI3Ks, with IA PI3K being the type most widely implicated in human cancers [13]. Class IA PI3K primarily phosphorylate phosphatidylinositol-4,5,bisphosphate [PI(4,5)P2] in the plasma membrane to generate the second messenger phosphatidylinositol-3,4,5,trisphosphate [PI(3,4,5)P3]. These enzymes are heterodimers, consisting of a p85 regulatory and p110 catalytic subunit [13]. Class IA PI3K are most often activated by RTK signaling and indirectly by Ras. Upon RTK signaling, p85 binds to either phosphotyrosine residues or adaptor molecules. This binding serves both to recruit the p85-p110 heterodimer to the plasma membrane and to relieve the basal inhibition of p110 by p85. p110 then phosphorylates PI(4,5)P2 to generate PI(3,4,5)P3. The 3-phosphatase PTEN (phosphatase and tensin homologue) dephosphorylates PI(3,4,5)P3 and catalyses the reverse reaction. PI(3,4,5)P3 binds a subset of pleckstrin homology domain-containing proteins, including 3-phosphoinositide-dependent protein kinase (PDK1) and protein kinase B (also called AKT) to the plasma membrane. Once there, AKT is phosphorylated at Thr308 by PDK1 and Ser473 by the mammalian target of rapamycin (mTOR) complex 2 (mTORC2) [14]. It is believed that AKT is the central signal mediator of the canonical PI3K signaling pathway. However, recent studies also suggest that the link between PI3K and AKT can be uncoupled [15–18] and that oncogenic PI3K signaling can be transmitted through an AKT-independent pathway, further adding to the complexity of PI3K signal transduction. AKT phosphorylates numerous downstream targets that regulate a wide array of cellular processes important in tumor development and progression [19]. One of its major effectors is mTOR complex 1 (mTORC1), which is activated in multiple human cancers and is one of the major targets in the PI3K pathway for chemoprevention and therapy [14] (Figure 1).

In human HNSCC, molecular alterations at the levels of both expression and function have been identified. These include gain-of-function mutations and amplifications in *PIK3CA* (the gene coding p110*α*, the catalytic subunit of PI3K), loss of heterozygosity and inactivating mutations in *PTEN*, and overexpression/activation of AKT and mTOR signaling [5, 6]. Several reports utilizing the GEMM approach, including our own studies, have confirmed the functional importance of these molecular alterations in HNSCC development and progression. In the following sub-sections, we will summarize several molecular alterations and functional studies, particularly those using GEMMs, involving molecular components of the PI3K pathway in HNSCC tumorigenesis.

### 3.1. PIK3CA

*PIK3CA*, the gene coding for the catalytic subunit p110*α* of PI3K, is one of the most commonly mutated oncogenes in multiple human malignancies (3441 mutated samples among a total of 27725 samples, about 12%, according to the Catalogue of Somatic Mutations in Cancer (COSMIC) Database (http://www.sanger.ac.uk/genetics/CGP/cosmic/)). Most of these mutations are clustered in exon 9 and exon 20, which corresponds to the helical domain mutant E545K, and the kinase domain mutant H1047R, respectively. Almost all *PIK3CA *mutations are gain-of-function mutants, further supporting its oncogenic role in human malignancies [1]. In human HNSCC samples,* the PIK3CA* mutation rate is about 10% [20] but is relatively higher (20%) in HNSCC arising from a pharyngeal site [21]. In addition to somatic mutations, genomic amplification of *PIK3CA* has also been reported in several human cancers [1]. Interestingly, a significantly higher percentage of *PIK3CA* gene amplification was noted in squamous cell carcinoma, compared to adenocarcinoma in lung [22]. In human HNSCC tissue samples, over 30% of cases involving *PIK3CA* amplification involve the candidate gene residing in the common amplification region of 3q26.3 in human HNSCC samples [23, 24]. 

*PIK3CA* alterations have been associated with cancer recurrence [25], metastasis [26, 27], and poor prognosis [28, 29] in a variety of human cancers. In HNSCC, *PIK3CA* alterations correlate with an advanced stage [30, 31], vascular invasion [24], and lymph node metastasis [32]. Interestingly, in breast cancer cell motility and metastatic potential are differentially enhanced depending on whether their mutations are localized at the helical or kinase domain.* An* overexpression of the helical domain through mutation E545K of *PIK3CA *produces a more severe metastatic phenotype compared to that of the kinase domain mutation H1047R [33]. 

Although the activated forms of *PIK3CA,* generated through either mutation or amplification, are transforming in vitro [1, 13], their oncogenic potential in vivo has only recently been assessed through the GEMM approach. While deletion or inactivation of *PIK3CA *significantly impairs oncogenic transformation [34] and produces a significant resistance to Ras-oncogene-induced tumorigenesis [35], overexpression of *PIK3CA* results in hyperplasia in ovarian surface epithelium [36] and can predispose mammary glands to neoplastic transformation [37]. Moreover, a knock-in *PIK3CA* H1047R mutant is sufficient to induce lung and breast cancer development [38–40]. Work being done in our own lab suggests that these trends apply to HNSCC as well. Using a HNSCC inducible transgenic mouse line that we had previously developed [9], we observe a strong oncogenic role of *PIK3CA *in HNSCC at both initiation and progression when it is overexpressed in head and neck epithelium (L. Du et al.'s manuscript in preparation).

The underlying molecular mechanisms of PI3K-driven oncogenesis are still unclear. Although AKT is largely regarded as the dominant mediator of oncogenic PI3K signaling [2], recent studies suggest that the link between PI3K and AKT can be uncoupled [15–18]. For example, PDK1, but not AKT, is activated in some breast cancers with *PIK3CA* mutations [15] (Figure 1). Using a mouse model of breast cancer conditionally expressing the *PIK3CA *H1047R mutant, Liu et al. have shown that *PIK3CA*-driven mammary tumors occur via both PI3K-pathway-dependent and PI3K-pathway-independent mechanisms, suggesting the complexity of the PI3K-driven oncogenic mechanisms [40]. To this point, sophisticated *PIK3CA-*GEMMs for a variety of cancer types may prove to be powerful tools in revealing the role of* PIK3CA* in a context- and stage-specific manner.

### 3.2. Other PI3K Molecules

Besides the common alterations of the *PIK3CA *gene encoding the catalytic p110*α* subunit of class IA PI3K, somatic mutations in the *PIK3R1* gene encoding the regulatory subunit p85*α* have been detected in multiple human cancers [41], including endometrium (26%), colon (5%), central nervous system (4%), breast (2%), pancreatic (2%), and skin (1%) (adapted from the COSMIC Database). Interestingly, somatic mutations of *PIK3R1 *are fairly common (7%, 3/41) in human HNSCC samples, with two missense mutations and one in-frame insertion [12]. The functional consequence of these mutants seems oncogenic as the mutants weaken an inhibitory interaction while retaining a stabilizing interaction between p85*α* and p110*α*, resulting in an activation of PI3K signaling [41]. However, p85*α* has also been shown to positively regulate PTEN [42], and reduced expression of p85*α* correlates with decreased PTEN expression [43]. Furthermore, deletion of *PIK3R1* in mouse liver resulted in aggressive hepatocellular carcinomas with pulmonary metastasis, suggesting a tumor suppressor role [43]. Thus, further characterization of *PIK3R1* mutants using both in vitro and in vivo approaches is warranted to reveal its role in HNSCC tumorigenesis. 

Similar to the oncogenic role of the p110*α* catalytic subunit, the other isoforms of the catalytic subunit, p110*β*, p110*γ*, and p110*δ*, have been shown to be oncogenic in experimental settings although there are no reports of molecular alterations of these isoform subunits in human cancer samples [44]. p110*β* has been studied the most among these isoform subunits, and most of the results came from the p110*β*-GEMM approaches. Using a *PTEN*-GEMM for prostate cancer, Jia et al. showed that ablation of p110*β*, but not p110*α*, impeded prostate tumorigenesis [45]. On the other hand, overexpression in a constitutively activated form of the p110*β* isoform induced prostate intraepithelial neoplasia in mice [46]. Furthermore, knock-in of a catalytically inactive form of p110*β* blocked tumor development in an ERBB2-GEMM for breast cancer [47]. Future research, using both human samples and GEMM approaches and aiming to assess the roles of these isoform subunits in HNSCC, will surely produce interesting results.

### 3.3. PTEN

PTEN acts as a negative regulator for the PI3K signaling by dephosphorylating PI(3,4,5)P3 and is the second most commonly mutated tumor suppressor in human cancers [48]. It is estimated that the overall frequency of *PTEN* mutations in sporadic human cancers is about 12% (2044 mutated samples among a total of 17452 samples according to the COSMIC Database), with endometrium cancer displaying the highest frequency among those considered (38%, 690 mutated samples among a total of 1837 samples in the COSMIC database). Somatic mutation of *PTEN* in SCCs of the head and neck is about 3% (22 mutated samples among a total of 745 samples in the COSMIC database) but is higher in SCCs of skin (14%, 92 mutated samples among a total of 658 samples in the COSMIC database). Compared to the relatively less common somatic mutation rate, loss of PTEN expression was more common (~30%) in human HNSCCs [49]. Loss of heterozygosity at chromosome 10q near *PTEN* was detected in over 70% of the *PTEN*-mutated HNSCCs [50], suggesting an inactivation of a typical tumor suppressor. Promoter hypermethylation of *PTEN* has also been detected in multiple PTEN expression-lacking human cancers [51, 52]. This is, however, infrequent (~5%) in human HNSCC samples [53]. Nonetheless, loss of PTEN expression has been correlated with tumor prognosis and incorporated into the grading system used for human HNSCC patients [49, 54].

 The mechanisms driving the loss of PTEN expression in human cancers are still unclear. Mutations of *PIK3R1* and *PIK3R2* have been shown to affect PTEN stability [55]. Posttranscriptional regulation of PTEN by the developmental transcription factor GRHL-3 has been shown to correlate with PTEN loss in SCCs in both the skin and the head and neck [56]. Another potential mechanism for PTEN loss is through posttranscriptional regulation by recently discovered mRNAs, namely miR-21, miR-26a, and miR-106b-25, all of which have been identified as PTEN-targeting mRNAs [56–58]. We have shown recently that miR-9 level is positively correlated with PTEN level in human HNSCC cell lines [59]. PTEN expression level is also regulated posttranslationally. For example, the ubiquitin ligase NEDD4-1 has been shown to negatively correlate with PTEN level [60]. Whether NEDD4-1 overexpression accounts for a subset of PTEN loss in human HNSCC samples requires further investigation (Figure 1). 

Other molecules closely related to PTEN have also been found to be altered in multiple human cancers. PIP3 RAC exchanger 2a (P-REX2a) has been implicated as a PTEN-interacting protein and antagonizes PTEN in human cancers [61]. Similar to the phosphatase activity of PTEN in the PI3K/AKT signaling pathway, inositol polyphosphate 4-phosphatase type II (INPP4B) is able to suppress the PI3K/AKT signaling pathway and behaves as a tumor suppressor in at least breast and ovarian cancers [62]. Lastly, the PTEN pseudogene *PTENP1* has been shown to regulate PTEN level and acts as a tumor suppressor in human cancers [63] (Figure 1). 

 One of the major consequences of PTEN alteration is the activation of its main downstream targets AKT and mTOR, which are oncogenic in HNSCC tumorigenesis and are attractive targets for cancer therapies [48]. However, recent studies have identified several novel pathways downstream of PTEN. For example, the JNK signaling pathway has been found to be a functional target of PTEN and is significantly associated with PTEN loss [64]. Protein synthesis by the RNA-dependent protein kinase (PKR) and the subunit of eukaryotic translation initiation factor 2 (eIF2) phosphorylation pathway is also required for tumor suppression by PTEN [65]. These results generate potential therapeutic targets to act alongside targeting of the canonical PI3K signaling pathway.

Given the potent tumor suppressor role of PTEN in multiple human cancers, GEMMs possessing tissue-specific deletion of PTEN have been created to better understand PTEN in tumorigenesis [66]. The most striking *PTEN*-GEMM for human cancer is the prostate *PTEN* deletion model, in which mice with a single deletion of *PTEN* in prostate cells developed metastatic prostate cancer [66]. *PTEN* deletion also resulted in spontaneous tumor development in other organs, such as breast, lung, bladder, and skin, with a wide range of tumor onset pattern and penetrance [66]. Furthermore, *PTEN* deletion increases susceptibility of mice to the induction of lung cancer by the tobacco carcinogen NNK [4-(methylnitrosamino)-1-(3-pyridyl)-1-1-butanone] suggesting a role for PTEN in tobacco-induced tumor initiation [67]. Additionally, *PTEN *deletion enhances tumor development and progression in the presence of additional molecular changes, such as Ras and p53 [68, 69]. Using head-and-neck-specific GEMMs, we deleted *PTEN* specifically in the head and neck region and observed both premalignant lesions and tumor development. This head-and-neck-specific *PTEN*-GEMM can be utilized as a model for testing chemoprevention and therapeutic approaches targeting PI3K pathway (J. P. Shen et al.'s manuscript in preparation). 

### 3.4. AKT

The serine/threonine kinase AKT is the central mediator of the canonical PI3K pathway and mediates multiple cellular processes, including cell survival, proliferation, angiogenesis, metabolism, and protein translation through numerous downstream signaling proteins [19]. There are many publications covering almost every aspect of AKT relation to human cancers. For this particular paper, we will focus on the following topics: (1) molecular alterations of AKT in human cancers, (2) isoform-specific role of AKT in human cancers, (3) tobacco carcinogen-induced AKT activation, (4) in vivo role of AKT in human cancers, and (5) regulation of AKT by interacting proteins. 

#### 3.4.1. Molecular Alterations of AKT in Human Cancers

Given its central node position in the canonical PI3K pathway, AKT can be activated by either upstream *PIK3CA* activation or *PTEN* inactivation. This subsequent AKT activation, together with molecular alterations of AKT itself, represents one of the most frequent molecular changes in human cancers and provides rationale for targeting AKT as a therapeutic approach. 

All three isoforms of AKT, that is AKT1, AKT2, and AKT3, have been reported to be altered in various human cancers [70, 71]. Somatic mutation occurs most frequently in *AKT1* and almost exclusively manifests as the E17K mutation [72, 73]. This* AKT1* E17K somatic mutation was detected in about 5% of breast cancers and 3% of both thyroid and urinary cancers (adapted from the COSMIC database). Although the *AKT1* E17K mutation has not been identified so far in human SCCs of head and neck according to a single report [74], this mutation has been found in human SCCs of lung [75]. Mutations of *AKT2* have been sporadically reported in various human cancers, but none of these mutations occur at the corresponding position of the E17K in *AKT1 *[71]. Of particular note, somatic mutation of *AKT2* is relatively common in endometrial carcinoma [71]. Mutations of *AKT3* at both E17K and other sites were reported in melanoma [76] and endometrial carcinoma [71]. Unfortunately, as of yet there are no studies examining* AKT2* and *AKT3 *mutations in human HNSCC samples. It is also worth noting that, although several studies have shown the oncogenic properties of these *AKT *mutations in vitro, a confirmation of their functional significance requires further investigation and demonstration of validity in vivo. 

In addition to somatic mutation, overexpression of AKT isoforms, particularly AKT2, has been reported in multiple human cancers [77]. Gene amplification of *AKT2 *was reported in human ovarian and pancreatic cancers [77]. Also, overexpression of *AKT2* at the messenger level has been shown in breast and colon cancers and seems to correlate with cancer migration, invasion, and metastasis [78, 79]. Interestingly, *AKT2 *has been shown to be transcriptionally regulated by the master regulator of epithelial-mesenchymal transition (EMT), Twist, and is associated with tumor progression and metastasis [78, 79]. Overexpression of AKT1 and AKT3 has only been shown in human gastric cancer [77] and melanoma [80], respectively. In human HNSCC samples the overexpression of AKT2, but not AKT1 or AKT3, has been reported in one study [81].

Pan-AKT activation through phosphorylation of Ser437 and Thr308 is fairly common in multiple human cancers [19, 70]. Evaluation of these phosphorylation sites yields prognostic value in human lung cancer [82] and predicts chemotherapeutic benefit in breast cancer [83]. Persistent AKT activation is also common in human HNSCC samples, and occurs as early as the premalignancy stage, including dysplasia and carcinoma in situ, suggesting that AKT activation is an early event in human HNSCC tumorigenesis [6, 84, 85]. However, reports have also shown AKT activation to correlate with a poor clinical outcome in human HNSCC patients [86, 87]. The role of AKT activation in human HNSCC development and progression is still in need of further investigation. 

#### 3.4.2. Isoform-Specific Role of AKT in Human Cancer

Although AKT1, 2, and 3 share high sequence homology, clinical studies suggest the existence of isoform-specific roles of AKT in multiple human cancers [19, 70]. This is further validated by experimental studies using both in vitro and in vivo approaches. While AKT1 and AKT2 play a similar role in regulating cell survival and proliferation, they behave distinctly in their regulation of cell migration and EMT [88, 89]. For example, AKT1 knockdown induces cell migration and EMT in breast cancer cell lines, while AKT2 knockdown suppresses these behaviors [90]. This is further exemplified in breast cancer mouse models: while overexpression of AKT1 accelerates ErbB-2 mediated mammary tumorigenesis and suppresses tumor invasion [91], overexpression of AKT2 markedly increases the incidence of pulmonary metastases in breast cancer [92]. These data suggest that AKT1 acts as a metastasis suppressor, while AKT2 as a metastasis promoter, further warranting the need to use isoform-specific AKT inhibitors in clinical management of cancer patients. 

The underlying mechanisms regulating the isoform-specific roles of AKT are still unclear. Distinct downstream targets of each AKT isoform might mediate this separate signaling transduction and be responsible for the distinct behavior of AKT isoforms in human breast cancer progression and metastasis. A recent report showing regulation of mRNA-200, which plays a critical role in cell migration and EMT, by the ratio of AKT1 to AKT2 [93] suggests another potential mechanism for the isoform-specific roles of AKT. Whether these trends are context or stage specific is still unclear. As of yet, there are no human HNSCC studies that address these questions though they are needed to guide future clinical trials on HNSCC patients using AKT isoform-specific inhibitors. 

#### 3.4.3. Tobacco Exposure and AKT Activation

Though tobacco exposure is one of most important etiological factors in HNSCC tumorigenesis, its underlying molecular mechanisms remain poorly understood [4]. In addition to the formation of DNA adducts, tobacco carcinogens, such as NNK, activate several signal transduction pathways, including AKT, in both normal and cancer cells in the lung [94]. We have shown that, in both HNSCC tumors and the adjacent mucosa, AKT is activated at a higher frequency in HNSCC patients who are smokers compared to those who are nonsmokers [95]. Also, adding physiologically relevant concentrations of NNK to normal head and neck epithelial cells and HNSCC cell lines will rapidly and constitutively activate AKT in a dose-dependent and time-dependent manner. Finally, we demonstrated that NNK exposure to mouse head and neck epithelium results in epithelial hyperproliferation and reduced apoptosis, which is correlated with AKT activation [95]. These studies suggest that AKT activation plays a pivotal role in mediating tobacco-induced HNSCC carcinogenesis and that it may be an effective target for chemoprevention.

#### 3.4.4. In Vivo Role of AKT in Human Cancers

The precise functional consequence of AKT activation cannot be assessed without in vivo studies. This is particularly critical for the evaluation of the isoform-specific role of AKT in context- and stage-specific manners. Current in vivo models of AKT activation overwhelmingly confirm its oncogenic role at differing levels of potency in various cancer types. However, at least in breast cancer models, the distinct roles of different AKT isoforms in cancer progression and metastasis have been observed as described previously. Mice constitutively overexpressing an activated AKT driven by a keratin 5 promoter developed both skin and head and neck tumors and an increased sensitivity to skin carcinogenesis [96, 97]. The oral lesions seen in these specimens were mostly epithelial dysplasia, their malignant transition hampered by the induction of premature senescence. This suggests that AKT activation is an early event but not sufficient by itself to HNSCC tumorigenesis [97]. This is further supported by the introduction of p53 loss, which synergizes with AKT activation to develop metastatic HNSCC [97]. 

#### 3.4.5. Regulation of AKT by Interacting Proteins

Numerous proteins similar to PTEN have been identified as regulators of AKT activation and stability. PH domain leucine-rich repeat protein phosphatase (PHLPP) has been shown to attenuate AKT signaling through the regulation of distinct AKT isoforms [98]. Deletion or loss of expression of PHLPP has been reported in a significant fraction of colon and prostate cancers [99, 100]. FK506-binding protein 51 (FKBP51) has been shown to act as a scaffolding protein for AKT and PHLPP, as well as promote the dephosphorylation of AKT. Furthermore, FKBP51 is downregulated in pancreatic cancer samples and cell lines [101]. Posttranslational modifications of AKT through ubiquitinating proteins such as TTC3 have also been reported [102]; however, this protein role in oncogenic AKT activation has not been studied. Lastly, the p53 target gene PH domain-only protein (PHLDA3) has been found to compete with the PH domain of AKT for binding to membrane lipids, thereby inhibiting AKT translocation to the cellular membrane and, thus, its activation. Consistent with its function, loss of the PHLDA3 genomic locus is frequently observed in primary human lung cancer samples [103]. However, such studies have not yet been reported in HNSCC cases (Figure 1). 

### 3.5. PDK1

Although it is widely accepted that AKT activation acts as the primary oncogenic mediator in canonical PI3K signaling [2], as has been stated previously in this paper, recent studies suggest that the link between PI3K and AKT can be uncoupled [15–18]. For example, AKT signaling is diminished in human breast cancer cell lines and clinical samples harboring *PIK3CA *mutation. In lieu of AKT, these cells make use of a signaling pathway involving the PI3K effector PDK1 and its downstream substrate SGK3 [15]. Compared to AKT, there are few studies of PDK1 in human cancers. Increased gene copy numbers of *PDK1 *have been found in 21% of breast cancer samples, and total PDK1 mRNA and protein have been observed to be overexpressed in the majority of human breast cancer [104]. Overexpression of PDK1 promotes invasion and activation of matrix metalloproteinase [105], while downregulation of PDK1 inhibits migration and experimental metastases of human breast cancer cells [106]. Introduction of a hypomorphic mutation of* PDK1 *in a *PTEN *cancer mouse model suppresses tumorigenesis [107], confirming the oncogenic role of PDK1 as one of the downstream effectors of the PI3K/PTEN signaling, and suggesting PDK1 as a promising anticancer target. However, the role of PDK1 and its correlation with the canonical PI3K/PTEN/AKT pathway in HNSCC development and progression has not been assessed yet. 

### 3.6. mTOR and Its Related Molecules

Among the numerous molecules that could act as downstream effectors of the PI3K/AKT pathway, mTOR is of particular interest. mTOR assembles into at least two distinct complexes, that is, mTORC1 and mTORC2. mTORC2 contains Rictor, SIN1 and mLST8/GbL and acts upstream of AKT to phosphorylate the Ser473 of AKT. In contrast, mTORC1 contains Raptor, PRAS40, and mLST8/GbL and acts as a major downstream target of AKT. mTORC1 regulates cell growth by controlling key eukaryotic translational regulators, including p70-S6 kinase, and the eukaryotic translational initiation factor, 4E binding protein 1 (4E-BP1) [14]. In addition, the growth factor receptor-bound protein 10 (Grb10) has been recently identified as an mTORC1 substrate. The mTORC1-mediated phosphorylation stabilizes Grb10, leading to the inhibition of the PI3K and ERK-MAPK pathways. Interestingly, Grb10 is frequently downregulated in various human cancers, with the loss of Grb10 and PTEN being mutually exclusive. This indicates Grb1 as being a tumor suppressor, potentially regulated by mTORC1 [108] (Figure 1). 

In addition to directly activating mTORC1, AKT also phosphorylates tuberous sclerosis complex (TSC) 1 and TSC2, releasing their inhibition of the Ras-like small G protein, Rheb, which in turn activates mTORC1. During conditions of low nutrient availability, mTOR signaling is normally inhibited by AMP activated protein kinase (AMPK), which is activated by its upstream serine/threonine kinase LKB1 (Figure 1). Interestingly, these negative regulators of mTOR, that is, TSC1, TSC2, and LKB1, are tumor suppressors, and germline mutations of *TSC1/2* or *LKB1 *cause hamartomas and predisposition to multiple malignancies in humans [14]. The critical connection of mTOR to the PI3K/AKT pathway has led to the prediction that the targeting of mTOR may be useful in cancer therapy. Indeed, using the mTOR inhibitor, rapamycin, yields promising results for multiple human cancers [109]. 

In human HNSCC, activation of mTOR/p70-S6/4E-BP1 pathway is a frequent event in clinical specimens and cell lines [110]. Amplification of Rictor has been reported in one study [111]. LOH of *TSC1/2* and DNA methylation of *TSC2* have been reported in human HNSCC samples [112], and overexpression of TSC2 inhibits cell growth both in vitro and in vivo [113]. A somatic mutation of *LKB1*, which leads to the loss of growth inhibition, has been found in a human HNSCC patient [114]. Decreased nuclear LKB1 levels have been shown to correlate with HNSCC metastasis [115]. Consistent with the effects of anti-mTOR therapy in other cancers, inhibition of mTOR by rapamycin displays a potent antitumor effect in HNSCC in vitro [110], in oral carcinogenesis model [116], and in a HNSCC-GEMM [117]. Targeting mTOR has also been shown to be a possible adjuvant therapy for microscopic residual disease in human HNSCC patients [118]. However, studies exploring the frequencies of these molecular alterations and their mechanisms in HNSCC tumorigenesis are still lacking. 

## 4. PI3K Pathway as Target for Chemoprevention and Therapy

### 4.1. Chemoprevention

Activation of PI3K/AKT/mTOR pathway has been illustrated as an early event in multiple human cancers, suggesting that targeting the PI3K/AKT/mTOR pathway may have chemopreventive value. This is further exemplified by tobacco exposure activation of AKT/mTOR in multiple tobacco-related malignancies, including HNSCC [95, 119]. The tobacco-associated NNK and the DNA adduct-forming agent 4-nitroquinoline-1-oxide (4NQO) activate AKT/mTOR as early as premalignancy stage [95, 119, 120]. Inhibition of this pathway by either Deguelin, a natural compound belonging to the rotenoid family [121], or Metformin, an antidiabetes medicine, has been shown to possess chemopreventive effects on a tobacco carcinogen-induced lung tumorigenesis model [122]. In addition, mTOR inhibition by rapamycin has been shown to prevent early onset of HNSCC tumorigenesis in both the 4NQO-induced HNSCC mouse model [116] and a HNSCC-GEMM [117]. Additionally, resveratrol, a phytoalexin enriched in red grapes, strawberries, and peanuts, has been found to be a potent chemoprevention agent for many cancers [123]. One of the major mechanisms of its chemopreventive effect is through inhibition of the PI3K/AKT/mTOR pathway [124]. It will be interesting to study its chemopreventive effect on both the 4-NQO-induced HNSCC mouse model and the HNSCC-GEMMs. 

### 4.2. Targeted Therapy

Personalized cancer therapies with selective molecular targets have emerged as a novel class of anticancer agents, with demonstrated clinical efficacy and less toxicity than conventional therapies [125]. In this situation, the PI3K/AKT/mTOR pathway has been extensively studied in almost all human malignancies including HNSCC and in both experimental and clinical settings [126]. Multiple drugs have been designed to target this pathway, making it the most “druggable” pathway for targeted therapies of human cancers. This has been summarized and reviewed in many articles. Given the scope of this paper, we will only comment on a few aspects. For a more detailed explanation of progress in PI3K targeted therapy on HNSCC, please refer to any of the several excellent reviews available on the topic [3, 5, 126, 127].

**(1) Identification of Biomarkers for the Personalized Cancer Therapy Targeting the PI3K Pathway**

Since the concept of personalized cancer therapy is based on the identification of a subset of patients whose tumors carry specific molecular alterations, biomarker identification is critical for predicting the effectiveness of targeted therapy [125]. For example, both *PIK3CA *mutations and nuclear phosphorylation of AKT are shown as biomarkers for the effectiveness of PI3K inhibitors for human cancer patients [128, 129]. In addition, human cancer patients harboring PIK3CA mutations are sensitive to targeted therapy using the mTOR inhibitor everolimus, while human cancer patients carrying *Kras* mutations are resistant to the treatment [130]. This is further confirmed in a *PIK3CA*-GEMM and *Kras*-GEMM for lung cancer. While NVP-BEZ235, a dual pan-PI3K and mTOR inhibitor, led to marked tumor regression in the PIK3CA-GEMM, it did not affect tumor growth significantly when treating the Kras-GEMM unless it was used in combination with a MEK inhibitor [38]. Finally, a recent report of the screening of over three hundred nonredundant PI3K-pathway-relevant phosphopeptides identified PRAS40, a molecule involved in protein phosphorylation, as a biomarker correlated with PI3K pathway activation and AKT inhibitor sensitivity [131].

**(2) Activation of PI3K/AKT/mTOR Pathway as a Resistance Mechanism to Targeted Therapy or Radiotherapy**

EGFR targeted therapy is the first FDA-approved protocol for treating human HNSCC patients [132]. However, resistance to EGFR therapy remains a major obstacle to positive clinical outcomes [127]. Activation of the canonical PI3K/AKT/mTOR pathway seems to be associated with resistance to EGFR inhibitor in multiple human cancers [133]. However, a recent study showed that an EGFR-activating mutation resistant to targeted therapy activates the mTORC2-NF-*κ*B signaling pathway in an AKT-independent manner in glioblastoma patients [134]. Further studies are necessary to investigate the role of both canonical and noncanonical PI3K pathways in resistance to EGFR therapy in human HNSCC patients. HNSCC is relatively sensitive to radiotherapy [135]. However, activation of the PI3K/AKT/mTOR pathway is implicated in all major mechanisms of radioresistance, including intrinsic radioresistance, tumor cell proliferation, and hypoxia [135]. Thus, blocking the PI3K/AKT/mTOR pathway has great potential to enhance the effectiveness of radiotherapy for HNSCC patients.

**(3) Synergistic Effect of Combination with Other Receptor Tyrosine Kinase Targeted Therapies**

Recent evidence of multiple feedback loops and interactions with other signaling pathways highlights the complexity of PI3K signaling. Using an inducible *PIK3CA*-GEMM for breast cancer, Liu et al. identified c-Myc elevation as a potential mechanism by which tumors develop resistance to PI3K-targeted therapies [40]. Moreover, inhibition of AKT induces activation of upstream RTK signaling pathways, such as HER3 [136], and mTOR inhibition causes activation of AKT signaling [137] or MAPK pathway [138]. These studies suggest that combination therapies of PI3K-targeted therapy together with targeting c-Myc, Her3, or MAPK pathway, may be more effective for the treatment of certain human cancer patients.

## 5. Prospectus

Mounting evidence clearly shows both the paramount importance of the PI3K pathway in the tumorigenesis of many human malignancies including HNSCC, and the promising results of targeting this pathway for treatment of human cancer patients. However, there are still many questions that need to be answered. Compared to the extensive studies on PIK3CA, there are few studies on the other subunits of class IA PI3Ks and their interactions with PIK3CA and PTEN. Studies of classes II and III of PI3Ks in human cancers are generally lacking. Although several proteins interacting with PTEN or AKT have been shown to play a role in tumorigenesis, more studies must be undertaken to discover novel molecules modulating the PI3K pathway, and assess their roles in tumorigenesis. In addition to the relatively linear canonical PI3K/AKT pathway, more and more noncanonical pathways are expected to be identified. Furthermore, the newly discovered mRNAs described in this paper add yet another layer of complexity to our understanding of the molecular regulation of the PI3K signaling pathway. Integrative mapping of molecular alterations in human cancers, particularly in HNSCC samples, is highly demanding. Utilization of multiple molecular approaches, especially GEMMs of the PI3K signaling pathway, will help us to better understand the complexity of this pathway in human cancers, as well as in context and stage-specific manners. Ultimately, these studies will yield identifiable biomarkers for improved clinical diagnosis and prognosis, contributing to strategies of therapy and prevention that will allow for the better management of human cancers and better outcomes for human patients.
